# Double-Edged Sword: Exploring the Mitochondria–Complement Bidirectional Connection in Cellular Response and Disease

**DOI:** 10.3390/biology13060431

**Published:** 2024-06-11

**Authors:** Jingfei (Carly) Lin, Sinwoo (Wendy) Hwang, Honglin Luo, Yasir Mohamud

**Affiliations:** 1 Centre for Heart Lung Innovation, St. Paul’s Hospital, Vancouver, BC V6Z 1Y6, Canada; 2 Department of Pathology and Laboratory Medicine, University of British Columbia, Vancouver, BC V6Z 1Y6, Canada

**Keywords:** mitochondria, complement system, innate immunity, cell signaling, damage-associated molecular patterns (DAMPs)

## Abstract

**Simple Summary:**

Complement system is an ancient immune pathway that protects us against harmful invaders. Meanwhile, mitochondria, widely known as the powerhouse of the cell, are essential for producing energy and maintaining healthy cells. Bridging past and present studies, this review aims to elucidate an underexplored yet essential connection between the complement system and mitochondria. These interactions are implicated in different diseases across various tissues and organs, such as Alzheimer’s disease and cancer, that are in critical need of cure. Mitochondria release signals that impact the activation of the complement system, leading to tissue damage and inflammation; while the complement system alters mitochondrial functions, forming a vicious cycle. This cycle exacerbates disease progression and health outcomes. We hope to highlight potential targets to alleviate societal healthcare burdens by bringing scientists’ attention to this novel bidirectional interaction.

**Abstract:**

Mitochondria serve an ultimate purpose that seeks to balance the life and death of cells, a role that extends well beyond the tissue and organ systems to impact not only normal physiology but also the pathogenesis of diverse diseases. Theorized to have originated from ancient proto-bacteria, mitochondria share similarities with bacterial cells, including their own circular DNA, double-membrane structures, and fission dynamics. It is no surprise, then, that mitochondria interact with a bacterium-targeting immune pathway known as a complement system. The complement system is an ancient and sophisticated arm of the immune response that serves as the body’s first line of defense against microbial invaders. It operates through a complex cascade of protein activations, rapidly identifying and neutralizing pathogens, and even aiding in the clearance of damaged cells and immune complexes. This dynamic system, intertwining innate and adaptive immunity, holds secrets to understanding numerous diseases. In this review, we explore the bidirectional interplay between mitochondrial dysfunction and the complement system through the release of mitochondrial damage-associated molecular patterns. Additionally, we explore several mitochondria- and complement-related diseases and the potential for new therapeutic strategies.

## 1. Introduction

Mitochondria are dynamic organelles critical for key processes, including cellular energy production through oxidative phosphorylation (OXPHOS), metabolism, homeostasis, and cell signaling. Hence known as the powerhouse of the cell, mitochondrial dysfunction can lead to severe consequences. Impaired mitochondria have a causal relationship with several diseases, such as neurodegenerative disorders, metabolic syndromes, and cardiovascular diseases [1,2,3].

Interestingly, the endosymbiotic theory proposes that mitochondria share an evolutionary ancestry with bacteria. Hence, as remnants of ancient bacteria within host cells, the immune system may perceive mitochondria as foreign entities. Among the diverse immune responses, the complement pathway is widely recognized as a first-line defense against microbial infections. Comprised of over 30 secreted and membrane-bound proteins, the complement system serves diverse functions in both innate immunity and adaptive immune responses [4,5]. The complement system can detect pathogens by three main pathways to trigger a cascade of reactions, leading to inflammation, cell death, and pathogen elimination. In the last decade, advances in complement research have revealed novel functions of the complement system, including the intracellular complement system, known as the complosome [6,7]. The complosome has been found to play a central role in metabolism and cellular homeostasis, modulating essential processes in mitochondria such as glycolysis, ATP generation, and OXPHOS [8,9,10,11]. Considering the revelations, further investigation of the implications in cellular mechanisms and disease is needed. While the term “complosome” is specifically employed to denote intracellular complement, this review refrains from making a clear differentiation between intracellular and extracellular complement elements. Given the recent establishment of the complosome concept, distinctions between these compartments remain to be further characterized.

In the past 30 years, novel insights have revealed a tight relationship between the complement system and mitochondria. The complement system employs pattern recognition receptors to detect damage-associated molecular patterns (DAMPs) released from infected, injured, or stressed cells, signaling an immune response [12,13,14,15]. Under stress, mitochondria release DAMPs, triggering activation of the innate immune system, which can result in heightened oxidative stress and additional damage to mitochondria, leading to a destructive cycle [16,17,18,19]. However, the impact of this interaction on disease pathogenesis is underexplored. Exploring the complex relationship between the complement system and mitochondria in various diseases will help reveal potential therapeutic targets. This review aims to provide an in-depth examination of the reciprocal relationship between mitochondrial dysfunction and the complement system, with a particular focus on their implications in immune signaling and their role in disease pathogenesis.

## 2. Mitochondria

Mitochondria play pivotal roles in diverse cellular processes, including ion homeostasis and programmed cell death/apoptosis. When subjected to stressors such as genetic mutations, viral infections, or aging, mitochondria are damaged and activate quality control mechanisms to maintain homeostasis. Mitochondria are constantly under dynamic changes through fission and fusion processes, allowing for the removal of damaged mitochondrial segments and the exchange of components [20,21,22]. Mitophagy is another important pathway whereby autophagosomes selectively remove damaged or dysfunctional mitochondria [23]. Alternatively, the mitochondrial unfolded protein response induces protein folding and eliminates damaged components [24,25].

Mitochondria release DAMPs in response to cellular distress. Under ideal circumstances, quality control processes effectively mitigate stressors, so mitochondrial release of DAMPs would be minimized. However, under extreme stress, when quality control processes prove insufficient, mitochondria release DAMPs such as cytochrome c, reactive oxygen species (ROS), mitochondrial DNA (mtDNA), cardiolipin, and ATP [26,27,28]. DAMPs can also induce numerous innate immune responses [29]. Furthermore, extracellular accumulation of ATP can lead to loss of mitochondrial membrane potential, disrupting ion homeostasis and cellular bioenergetics [26]. This impairment affects processes reliant on energy availability, such as active transport, cellular signaling, and maintenance of membrane potential [26,29]. Additionally, the release of mtDNA can be recognized by cyclic GAMP-AMP synthase/cGAS, triggering inflammation through the type-I interferon (IFN) pathway [30]. In summary, while mitochondria employ quality control processes to maintain homeostasis, profound mitochondrial damage leads to disrupted ATP production, overproduction of ROS, and cell death. This results in DAMP release to induce innate immune responses. Hence, understanding the ramifications of mitochondrial dysfunction is crucial for developing targeted therapies.

## 3. Complement System

**Figure 1:**
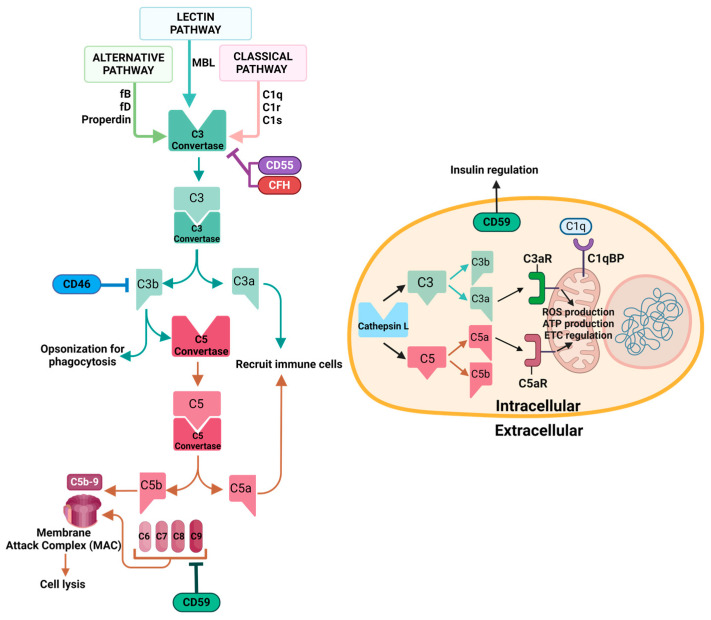
**The extracellular and intracellular complement system.** The complement system is activated by three main pathways: the classical pathway via C1q, C1s, and C1r forming the C1 complex; the lectin pathway via mannose-binding lectin (MBL); and the alternative pathway via spontaneous hydrolysis of C3, involving factor B (fB), factor D (fD), and properdin. All three pathways converge on the formation of the C3 convertase, which activates C3 by cleaving it into C3a and C3b. C3b, along with other complement components, forms the C5 convertase, which activates C5 by cleaving it into C5a and C5b. The cascade of reactions leads to the formation of the membrane attack complex (MAC). Regulators of the complement system (such as complement factor H (CFH), CD55, CD59, CD46) inhibit different steps of the cascade as indicated. Intracellular CD59 also has a role in insulin regulation. C1q, C3a, and C5a bind to receptors located on mitochondria, regulating mitochondrial functions.

The complement system is part of the innate immune system. The extracellular complement system is activated through three main pathways: classical, alternative, and lectin pathways. Although each pathway is initiated by different signaling processes, they all converge on the formation of complement component 3 (C3) convertase, which activates C3 by cleavage. Activation of C3 produces building blocks for complement component 5 (C5) convertase, which in turn activates C5. Additionally, activation of complement proteins produces opsonins (e.g., complement component 1q (C1q), C3b, inactivated C3b (iC3b), C3d) and anaphylatoxins (e.g., C3a and C5a) that opsonize pathogens for phagocytosis and recruit immune cells to the site of activation, respectively [4,31]. Ultimately, activation of the complement system leads to the formation of a membrane attack complex (MAC), a pore structure on the cell membrane that causes osmotic imbalance, leading to cell lysis. Recent discoveries of the complosome have demonstrated non-canonical functions of the complement system apart from its canonical functions in immune defense, including opsonization, inflammation, and cell lysis. A summary of the intracellular and extracellular complement system is shown in Figure 1.

### 3.1. Internalization Mechanisms of Complement Proteins

Complement proteins are not only produced intracellularly but can also be re-ingested and retro-translocated into the cell. For instance, King et al. found that C3 contains alternative ATG start codons, enabling its translation in the cytosol [32]. They discovered that this form of C3 loses the ability to be secreted and can rescue autophagy in cells with C3 knockout, whereas uptake of extracellular C3 does not achieve this. Additionally, C3 can be produced and secreted, then taken up again by the same cell. For example, Kremlitzka et al. demonstrated that alternative translation start sites of C3 result in the production of the cytosolic form of C3 without glycosylation, exhibiting distinct functions compared to the serum form [33]. The same study also showed that intracellular C3 is retro-translocated from the ER to the cytosol [33]. Similarly, Golec et al. suggested that CD59 is retro-translocated from the ER to the cytosol, and that cytosolic CD59 lacks a glycosylphosphatidylinositol (GPI) anchor [34]. While silencing CD59 resulted in dysregulated insulin production, removing the GPI anchor did not do so, suggesting the existence of an intracellular, non-GPI-anchored pool of CD59 that functions in insulin secretion [34]. Together, this evidence suggests that intracellular complement proteins such as C3 and CD59 have unique genetic and functional profiles, involving intracellular production, retro-translocation, and distinct roles in cellular processes, diverging significantly from their extracellular counterparts.

### 3.2. Intracellular Complement System

The complement system is predominantly recognized as a liver-derived and serum-circulating extracellular system. Although most complement factors are produced in the liver and secreted, C3 and C5 can also be produced and secreted by T cells [6,9]. Serum complement components can bind to receptors on the surface of T cells to induce effector function and intracellular C3 and C5 production [35,36]. Additionally, C3 and C5 can be activated intracellularly in T cells [9,37]. While the majority of complement is systemic, local production by specific cell types serves specific functions [37]. For example, C1q is mostly/only produced by immune cells including macrophages and dendritic cells [37,38,39]. Furthermore, C3a receptor (C3aR) and C5a receptor (C5aR) are present on intracellular lysosomal membranes and mitochondrial membranes [6,8,40,41]. These recent insights call for further research into cellular and mechanistic consequences of the complosome within intracellular processes.

Ample evidence demonstrates the presence of diverse complement proteins intracellularly, such as complement factor H (CFH), CD59 (also known as protectin), C3, and C5. CFH is a complement regulator that accelerates the decay of the C3 convertase and serves as a cofactor in the inactivation of C3. CFH was found to localize to lysosomes and acts as a cofactor for cathepsin L (CTSL) in cleavage of C3 into iC3b [42]. In addition, CD59, a GPI-anchored negative regulator of MAC, was found to have intracellular functions in insulin secretions [34].

Complement proteins such as C3 have been discovered intracellularly and can activate immune responses, such as flagging viruses for degradation, mitochondrial antiviral signaling (MAVS), and cytokine production [43]. Cytosolic C3 was reported to interact with cytosolic proteins, such as ATG16L, a component of the autophagy mechanism that converts LC3-I to LC3-II, resulting in an autophagy-dependent restriction of bacterial growth [32,44]. Liszewski et al. demonstrated that C3 is activated intracellularly by CTSL, independent of C3 convertases in T cells [6]. It has also been suggested that C3 is stored in the endoplasmic reticulum and lysosomes in T cells, and the cleavage fragment C3a has a receptor on the lysosome. Upon C3a binding to C3aR, lysosomes can activate glycolysis and OXPHOS, leading to IFN production and activation of both T helper and cytotoxic T cells [6,8]. Additionally, significant levels of intracellular C5 and C5a have been shown by Niyonzima et al. [45]. The distinct localization of C5 and C5a may indicate that C5 activation occurs in post-Golgi compartments [45,46]. Furthermore, Daugan et al. revealed a novel intracellular function of C1s, a proteolytic enzyme that activates C4 and initiates the classical complement pathway in tumor progression and T-cell activation [47]. While silencing C1s decreases cancer cell proliferation and increases T-cell activation, extracellular C1s does not affect the same parameters, suggesting an intracellular role of C1s in tumor cells [47]. Together, these findings strongly support the existence of an intracellular complement system and the broad range of novel functions of the complement system.

### 3.3. Interaction of Complosome with Mitochondria

Exploration of the intracellular complement system has unveiled a wide range of novel functions, particularly its interactions with cellular components. Recent evidence has demonstrated that complosome can mediate cellular homeostatic and metabolic processes, as well as immune cell activation through its interaction with mitochondria. For example, C3 cleavage fragments, such as C3a and C3b, can bind to their respective receptors, C3aR and CD46, to induce mitochondrial processes such as glycolysis and OXPHOS, which are needed for T-cell activation [6,8,48]. Furthermore, the C5 fragment, C5a, has a receptor on mitochondrial membranes, known as mitochondrial C5aR. This receptor was found to play a role in modulating mitochondrial function, such as reducing ATP production in favor of interleukin (IL)-1β generation [45]. This finding was supported by the observation that IL-1β levels decrease after inhibition of intracellular C5aR. Most notably, C1q, one of the initiators of the classical complement pathway, binds directly to C1q binding protein (C1qBP) on mitochondrial membranes [1,49]. Interaction between C1q and C1qBP enhances the expression of mitochondrial biogenesis genes, optimizing respiratory function and oxygen consumption [50]. C1qBP has been identified as a key regulator in tumor progression, apoptosis, phagocytosis, and autoimmunity [1,51,52,53]. Evidently, diverse interactions between mitochondria and the complement system warrant further investigation. Uncovering these interactions will provide essential insights into their role in disease pathogenesis.

## 4. Role of Mitochondrial DAMPs in Complement Response

**Figure 2:**
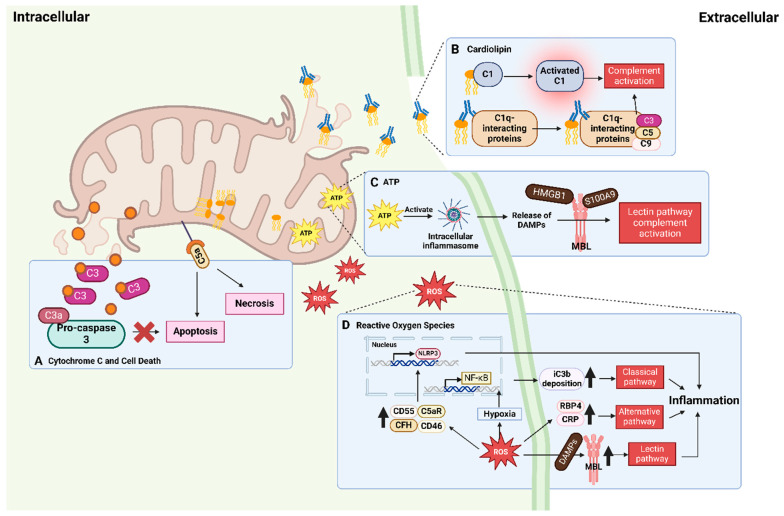
**Interactions between mitochondrial damage-associated molecular patterns (DAMPs) and the complement system.** Upon cellular stresses, mitochondrial DAMPs become mislocalized and are released from the mitochondria. (**A**) Cytochrome c interacts with C3, leading to the binding of C3a to pro-caspase 3, thereby inhibiting apoptosis. Conversely, binding of C5a to its mitochondrial receptor can induce apoptosis and necrosis. (**B**) Mislocalized cardiolipins from the inner mitochondrial membranes can bind to and activate C1, initiating complement activation. Anti-cardiolipin antibodies can also bind to C1q-binding proteins, providing a platform for the assembly of complement components, further resulting in complement activation. (**C**) ATP released into the cytosol activates intracellular inflammasomes, leading to further release of DAMPs, high molecular group box 1 (HMGB1), and S100 calcium-binding protein A9 (S100A9), which can be recognized by mannose-binding lectin (MBL) to activate the lectin pathway. (**D**) Reactive oxygen species (ROS) activate all three pathways of the complement system. Increased ROS levels elevate the expression of MBL, which recognizes DAMPs to activate the lectin pathway. ROS also induces elevated levels of inflammatory biomarkers such as retinol-binding protein 4 (RBP4) and C-reactive protein (CRP), which in turn activate the alternative pathway. Additionally, ROS enhances inactivated C3b deposition through the hypoxia-induced nuclear factor kappa B (NF-κB) pathway and promotes the expression of complement regulators and receptors, leading to inflammasome gene expressions and classical pathway activation. Receptors in the complement system, C5a receptor (C5aR), CD46, CD55, and complement factor H (CFH), are upregulated to promote the expression of NOD-, LRR-, and pyrin domain-containing protein 3 (NLRP3) to release proinflammatory substances.

This section will explore how mitochondrial DAMPs activate the complement system, linking mitochondrial damage with complement cascade as shown in Figure 2 and Table 1.

### 4.1. Intracellular Interactions and Impacts

#### Cytochrome C, Apoptosis, and Complement System

Cytochrome c, a molecule produced in mitochondria, facilitates ATP generation in the electron transport chain and regulates apoptosis [27]. It is widely recognized that under cellular stress, cytochrome c, which is initially localized on inner mitochondrial membranes, is released to the cytosol as a pro-apoptotic signal, triggering apoptosis through the activation of caspases [27]. Intriguingly, C3 and C5, which can also induce cell death, have been found to interact with cytochrome c intracellularly to modulate apoptosis [54,55]. For example, in the context of cardiac ischemia/reperfusion (I/R) injury, Fang et al. detected the presence of cytochrome c within the C3 complex inside cells [54]. Furthermore, under oxidative stress, direct interaction between C3 and cytochrome c in the cytosol was observed in the mouse model [54]. Conversely, in humans, Fang et al. found that C3 does not directly interact with intracellular cytochrome c. Instead, C3 binds to downstream elements of apoptosis, which is triggered by cytochrome c [54]. Additionally, the precursor of C3 was found to bind pro-caspase 3, an inactive component of apoptosis, to reduce apoptosis [54]. Despite the observed reduction in apoptosis by C3 and its precursor, it was noted that the interaction of C3 with the apoptotic pathway can promote necrosis, another type of cell death [54]. Therefore, this interaction between C3, cytochrome c, and apoptosis exerts opposing effects on cell death, increasing necrosis while decreasing apoptosis. Since I/R injuries can result from both necrosis and apoptosis, further understanding the degree of modulation of these two pathways by C3 will provide insights to mitigate damage.

In contrast, numerous studies have demonstrated that activated C5a anaphylatoxin exerts pro-apoptotic effects in myocardial I/R [55,56]. The use of anti-C5 treatment antagonizes C5 and decreases the formation of both C5a and MAC, effectively reducing the extent of necrosis and apoptosis in myocardial I/R by decreasing leukocyte infiltration and ROS generation [56]. Similarly, De Hoog et al. observed that the use of C5aR inhibitors leads to improved outcomes in myocardial I/R, evidenced by a decrease in the extent of heart tissue damage and a reduction in apoptosis [55]. Moreover, when C5aR is blocked or its expression is suppressed, leukocyte infiltration into the myocardial infarct area is decreased, thus reducing myocardial damage [55]. Hence, the protective benefits of C5aR inhibitors may result from preventing the binding of intracellular C5a to its mitochondrial receptor and subsequently blocking complement system activation, although the protective effects can also be a result of blocking the plasma membrane C5aR. Future studies are required to distinguish the effects.

### 4.2. Extracellular Interactions and Impacts

#### Cardiolipin and Complement System

Under cellular stresses, such as those encountered during ischemic events, cardiolipin, a phospholipid predominantly located in inner mitochondrial membranes, becomes accessible and/or mislocalized [57]. This results in structural and functional alterations to mitochondria, potentially triggering immunological responses including complement activation. Early investigations identified that cardiolipin located on mitoplasts (mitochondria lacking outer membranes) mediates activation of C1 extracellularly [58]. In instances of heart cell death, such as in ischemic heart disease, Rossen et al. discovered that the integrity of mitochondrial outer membranes is compromised, exposing inner membranes to the extracellular complement system [57]. These studies demonstrated that cardiolipin can bind to extracellular C1 and initiate the complement cascade, facilitated by negatively charged phosphate groups present in cardiolipin [57,58].

Furthermore, anti-cardiolipin antibodies were found to specifically accumulate in distinct regions of mitochondria, which are exposed to the extracellular space due to membrane ruptures in injured cardiac cells [57]. These antibodies were found to interact with over 15 mitochondrial components, several of which are proteins that bind to C1q [57]. Additionally, a specific group of C1q-interacting proteins was demonstrated to serve as stable sites for subsequent MAC formation [58]. Conversely, when mitochondria remain undamaged and unexposed to complement components, this interaction does not trigger a physiological response or further complement activation [58]. Therefore, cardiolipin released from the cell likely contributes to the propagation of complement-mediated inflammatory responses by binding to C1 and facilitating the assembly of subsequent complement components, underscoring its significant role in complement activation.

### 4.3. Intracellular and Extracellular Interactions and Impacts

#### 4.3.1. ATP and Complement System

Beyond its role as the energy currency of cells, ATP has an established role in immune modulation through its direct interaction with cells of the immune system. Studies have revealed that ATP, mostly generated in mitochondria via OXPHOS, can be released as a DAMP. ATP accumulates both intracellularly and extracellularly to trigger inflammation, particularly the lectin pathway of the complement system [5].

For hematopoietic stem/progenitor cells to be mobilized from the bone marrow into the bloodstream, mobilization is initiated by pro-mobilizing stimuli. Upon stimulation by these molecules, ATP produced in mitochondria is released to the cytosol, activating intracellular NOD-, LRR-, and pyrin domain-containing protein 3 (NLRP3) inflammasomes [59]. Ratajczak et al. discovered that ATP-induced inflammasome activation leads to the release of DAMPs, particularly extracellular high molecular group box 1/HMGB1 and S100 calcium-binding protein A9/S100A9. These released DAMPs were observed to bind to extracellularly circulating MBL, resulting in complement activation [59]. Following lectin pathway activation, pro-mobilizing stimuli also cause C5 activation, releasing C5 cleavage fragments, which can optimize the mobilization of stem cells from the bone marrow into peripheral blood [59,60]. The exact same mechanism of MBL-dependent complement activation was observed in microglia cells as well [61]. Hence, ATP not only plays a crucial role in energy supply but also acts as a potent activator of inflammation, through the activation of the lectin pathway in the complement system.

#### 4.3.2. ROS and Complement System

Mitochondria continuously generate ROS as byproducts of aerobic respiration. While ROS can serve as signaling molecules to activate the host defense system against pathogens, excessive ROS production can lead to oxidative damage [62]. Several prior studies indicate that oxidative stress damages cells, leading to the release of DAMPs which activates the extracellular complement system through all three pathways [17,63,64]. It was demonstrated that exposure of urban particulate matter to human umbilical vein endothelial cells enhances ROS production and subsequent activation of the complement system via the alternative pathway [64]. This observation was confirmed by inhibiting ROS production, which suppresses expression of retinol-binding protein 4/RBP4 and c-reactive protein/CRP, which are biomarkers of inflammation, indicating inhibited complement activation [64].

Interestingly, Collard et al. demonstrated that the process of hypoxia and reoxygenation in human endothelial cells triggers activation of the classical complement pathway, resulting in a notable increase in iC3b deposition [17]. Additionally, intracellular ROS can be released as an outcome of different types of cell death, including apoptosis, ferroptosis, and necroptosis, to stimulate extracellular complement activation and iC3b deposition [18,65]. In contrast, antioxidants and inhibition of nuclear factor kappa B (NF-κB) reduce iC3b deposition [18,65]. These findings suggest that iC3b deposition may be regulated by intracellular ROS-induced activation of NF-κB and activation of the classical complement pathway.

Furthermore, in vivo studies demonstrated elevated levels of rat mannose-binding lectin (MBL) and C3 deposition after myocardial reperfusion [63]. Conversely, following oxidative stress in endothelial cells in vitro, preventing accumulation of human MBL reduced complement system activation [63]. It is likely that stressed cardiac cells release DAMPs, which can bind to MBL, resulting in activation of the lectin pathway [66]. Increased expression of MBL also enables a higher possibility of complement activation upon release of DAMPs. Collectively, these findings demonstrate that the deposition of MBL and potential ROS-induced release of DAMPs can mediate the lectin complement pathway.

Dysregulation of oxidative species also leads to hypoxia and reoxygenation inside cells, resulting in increased expression of complement protective factors CD46 and CD55 on the surface of human umbilical vein endothelial cells [17]. Similarly, oxidatively stressed retinal pigment epithelial cells demonstrate elevated levels of complement receptors, such as C5aR, and complement component regulators such as CFH and properdin [67]. These regulators were observed to accumulate along with enhanced NLRP3 expression in retinal pigment epithelial cells under oxidative stress, leading to increased release of proinflammatory substances, regardless of external sources of complement [67].

### 4.4. Complement System Damages Mitochondria: A Vicious Cycle

As discussed earlier, mitochondria release DAMPs in response to cellular stress, modulating the activation of the complement system. Conversely, complement system activation can induce mitochondrial damage, leading to increased release of DAMPs. Thus, this interplay creates a self-perpetuating cycle that enhances complement system activation and amplifies immune responses. Activation of the complement system has been shown to trigger the production of ROS, causing tissue damage, highlighting the detrimental role of ROS [18]. Chronic ROS levels are known to induce oxidative damage to mitochondrial components, while acute ROS exposure can impair mitochondrial energy production, suggesting that complement-induced ROS likely disrupts mitochondrial structure and bioenergetic functions [16].

Furthermore, as discussed earlier, the pro-inflammatory effects of the complement system are well recognized. C3a and C5a induce the expression of inflammatory cytokines in immune cells, while sublytic MAC triggers the generation of additional inflammatory cytokines. Conversely, Choi et al. demonstrated that the presence of inflammatory cytokines, such as IL-1β or interferon-γ, leads to elevated levels of C3aR on the surface of human umbilical vein endothelial cells [64]. Additionally, complement activation on human umbilical vein endothelial cells following exposure to urban particulate matter stimulates the release of inflammation-inducing proteins, including c-reactive protein and retinol-binding protein 4, associated with the development of atherosclerosis and various cardiovascular diseases [64].

Beyond its known pro-inflammatory effects, recent studies have suggested a potential role of the complement system in metabolism and mitochondrial damage, particularly in regulating C3a and C5a. Nord et al. showed that blocking C3a signaling inhibits complement-induced inflammation and prevents mitochondrial damage, improving recovery from ischemic injury [68]. Similarly, Tsai et al. demonstrated that administration of C5a in kidney endothelial cells activates C5aR, resulting in ROS generation and apoptosis [69]. Inhibiting C5a reduces ROS production, cytochrome c release, and formation of apoptotic cells, suggesting that C5a-induced apoptosis is mediated through C5aR and directly impacts mitochondrial function.

The intricate interplay between mitochondria and the complement system underscores a self-perpetuating cycle wherein mitochondria release DAMPs, triggering complement system activation, and leading to further mitochondrial impairment. Understanding this interaction is crucial for developing treatments for mitochondria-related disorders.

**Table 1:** **Interactions between mitochondrial DAMPs and complement components.**

Mitochondrial DAMPs	Interactions with Complement	Reference
Cytochrome c	C3	[54]
	C5a	[55,56]
Cardiolipin	C1	[57,58]
ATP	MBL	[59,60]
ROS	iC3b	[17]
	MBL	[63]
	C5a	[69]

DAMPs: damaged-associated molecular patterns; MBL: mannose-binding lectin; ROS: reactive oxygen species.

## 5. Interplay of Complement and Mitochondria in Human Diseases

**Figure 3:**
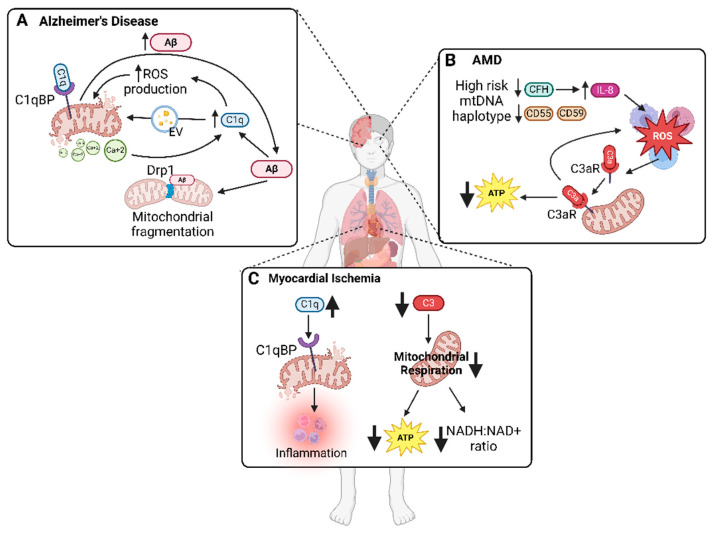
**Interactions between the complement system and mitochondria in localized diseases.** Complement system and mitochondria exhibit distinct interactions in various localized diseases, contributing to mitochondrial damage and complement dysregulation, thereby impacting disease progression. (**A**) In Alzheimer’s disease, amyloid-beta (Aβ) contributes to mitochondrial fragmentation via interaction with dynamin-related protein 1 (Drp1). Additionally, Aβ increases C1q levels, leading to the generation of ceramide-containing extracellular vesicles (EVs) and ROS, both of which contribute to mitochondrial impairments. Calcium overload resulting from mitochondrial damage further induces elevation in C1q levels. Subsequent binding of C1q to its mitochondrial receptor, C1q-binding protein (C1qBP), exacerbates Aβ levels, forming a vicious loop of dysfunction. (**B**) Age-related macular degeneration (AMD) is characterized by lower levels of complement regulators complement factor H (CFH), CD55, and CD59 in mtDNA haplotypes associated with high disease risk. Increased ROS leads to relocation of C3aR to mitochondria, leading to further ROS increase and ATP decrease. (**C**) Accumulated C1q at myocardial ischemic regions binds to mitochondrial protein C1qBP.

In addition to the mitochondrial release of DAMPs, diseases can result from diverse interactions between mitochondria and the complement system, particularly affecting high-energy-demanding organs. Interruptions in bioenergetics profoundly impact these vital areas. This section will explore how complement–mitochondrial damage interplay contributes to disease pathogenesis in localized and systemic conditions. The discussion on organ-specific diseases is summarized in Figure 3.

### 5.1. Alzheimer’s Disease

Alzheimer’s disease (AD) is the most diagnosed form of dementia and neurodegenerative disorder worldwide, yet effective treatments remain limited. Amyloid beta (Aβ), a crucial biomarker of AD, is known to damage mitochondria and activate the complement system, creating an intricate loop that propels the disease progression [70].

Numerous studies underscore mitochondrial dysfunction as a significant contributor to AD pathogenesis. For example, it has been observed that Aβ infiltration into mitochondria disrupts energy production at synapses, impeding neuron communication and resulting in cognitive decline [3]. Additionally, Aβ was demonstrated to interact with dynamin-related protein 1/DRP1, a mitochondrial protein responsible for mitochondrial fission. This interaction induces mitochondrial fragmentation and impairs mitophagy, leading to cognitive impairment in AD neurons [71]. Concurrently, Crivelli et al. found that Aβ triggers the release of proinflammatory factors, including C1q, which potentially activates the classical complement pathway in AD neurons [70]. They also observed that these proinflammatory factors induce the generation of extracellular vesicles enriched with ceramide, which plays a role in stimulating ROS production and disrupting mitochondrial membrane potential, thereby causing impairment of mitochondrial respiration [70,72]. Moreover, elevated C1q levels were shown to exacerbate mitochondrial damage through the induction of fatal oxidative stress in neurons [73]. Therefore, both Aβ and Aβ-induced C1q significantly impact mitochondrial functions, jeopardizing the energy supply required for proper neuronal functioning.

Reciprocally, in the early stages of AD, mitochondria damaged by Aβ were observed to subsequently induce increased Aβ accumulation [74]. This perpetuates a vicious cycle where elevated Aβ levels further impair mitochondrial functions, sustaining the progression of AD. Additionally, dysmorphic mitochondria often exhibit impaired or complete loss of homeostasis, including dysregulation of calcium signaling [75]. Datta et al. discovered that an overload of calcium can enhance C1q aggregation near dysmorphic mitochondria [76]. This phenomenon is likely due to the ability of calcium ions to stabilize C1q-like domains, thereby enhancing the accumulation of C1q around regions with elevated calcium concentrations [77]. Moreover, they showed that heightened C1q levels, resulting from calcium homeostasis disruption, trigger subsequent caspase-3 activation, leading to cell death [76,78].

Collectively, these studies support a disease model of AD pathogenesis characterized by the accumulation of Aβ, which damages mitochondria, leading to further Aβ accumulation and subsequent mitochondrial dysfunction. Therefore, given the current evidence from the literature, this cycle is likely to perpetuate the activation of C1q, thereby exacerbating mitochondrial damage and severe neurological symptoms. Escaping this detrimental cycle necessitates treatments that maintain mitochondrial functions and mitigate C1q and Aβ levels.

### 5.2. Age-Related Macular Degeneration

Age-related macular degeneration (AMD) is one of the major causes of irreversible vision loss in older age groups, impacting one in eight people 60 years of age or older [79]. The roles of complement proteins in retinal health and disease have been implicated. Trakkides et al. demonstrated that adult retinal pigment epithelial cells, or ARPE-19 cells, can produce complement proteins following oxidative stress [67]. Exposure to H_2_O_2_-induced oxidative stress results in elevated expression of complement receptors CR3 and C5aR1, as well as CFH in ARPE-19 cells [67]. Notably, CFH has been linked to an increased risk of AMD [80]. Furthermore, Kenney et al. concluded that the mtDNA haplotype protective against AMD expresses elevated levels of CFH compared to the haplotype at higher risk of AMD, suggesting a protective function of CFH in AMD [80]. Given that CFH inhibits C3 activation and degrades C3b, it may protect against AMD by lowering downstream complement activation, inflammation, and oxidative stress. This is further supported by enhanced CFH levels after exposure to T cells and oxidative stress [81,82]. In contrast to CFH, other inhibitors of the complement system, such as CD55 and CD59, were found to be downregulated after oxidative stress in ARPE-19 cells, making the cells more susceptible to complement-mediated damage [82]. This highlights intricate interactions between the complement system and oxidative stress in AMD, where CFH functions to inhibit upstream C3 activation and inactivate C3b, while CD55 and CD59 prevent downstream MAC formation.

Furthermore, the susceptible haplotype had decreased levels of ATP, suggesting differences in mitochondrial function, which plays an important role in AMD-associated retinal damage [80]. Together with the evidence that susceptible haplotypes had higher levels of CFH and C3, the complement proteins may play a role in altering mitochondrial processes, resulting in differences in ROS, ATP, and levels of inflammatory markers (i.e., IL-6, IL-1β, and tumor necrosis factor receptor 2/TNFR2) [83]. The production of IL-1β can be caused by C5aR and C3aR signaling via modulation of mitochondrial function and ROS production [45,84]. Notably, upon ROS exposure, C3aR relocates to mitochondria in APRE-19 cells, affecting calcium intake and ATP production [40]. Binding of C3a to mitochondrial C3aR results in decreased ATP production and increased calcium uptake and subsequently elevating ROS levels [85]. Collectively, these findings suggest that inhibiting C3 activation, and thus the formation of C3a offers protection to retinal pigment epithelial cells, at least in part through the mitochondrial C3aR mechanism.

Collectively, this evidence supports a connection between the complement system and mitochondria in AMD, especially through alterations of regulators of complement activation by oxidative stress. Understanding the intricate balance between complement regulation and mitochondrial ROS production is crucial for developing novel therapeutic interventions for AMD.

### 5.3. Myocardial Ischemia

Myocardial infarction (MI), occlusion of blood flow to the heart, is one of the main causes of cardiovascular mortality. MI-induced heart failure is associated with high morbidity and mortality [86,87]. Due to the large energy demand of the heart, mitochondria comprise a third of cardiomyocytes and provide 95% of ATP in the myocardium [88]. Both mitochondria and the complement system have been implicated in myocardial infarction. As early as 1975, Pinckard et al. identified the release of subcellular fragments in the heart that activate the complement system, triggering inflammation and diseases associated with MI [89]. This study revealed that these subcellular fragments responsible are likely mitochondria in origin, as supported by activation of C1, C2, C3, and C4 by the human heart mitochondria, and an interaction between complement C1 and mitochondrial membrane [89]. Subsequent studies have confirmed this mitochondria–complement protein interaction, especially between C1q and mitochondrial proteins [90,91,92,93,94]. In contrast, Torp et al. found that intracellular C3 protects the heart against I/R injury by promoting mitochondrial respiration [93]. Mice deficient in C3 show reduced mitochondrial respiration, as evidenced by lower levels of ATP and decreased NADH/NAD+ ratio compared to wild-type mice [93]. Furthermore, acute MI can induce biochemical and metabolic disruptions that trigger a series of reactions in mitochondria, resulting in calcium overload and mitochondrial dysfunction [95]. I/R injury could be attributed to the release of mitochondrial DAMPs, activating immune systems such as complement pathway [8,93].

**Figure 4:**
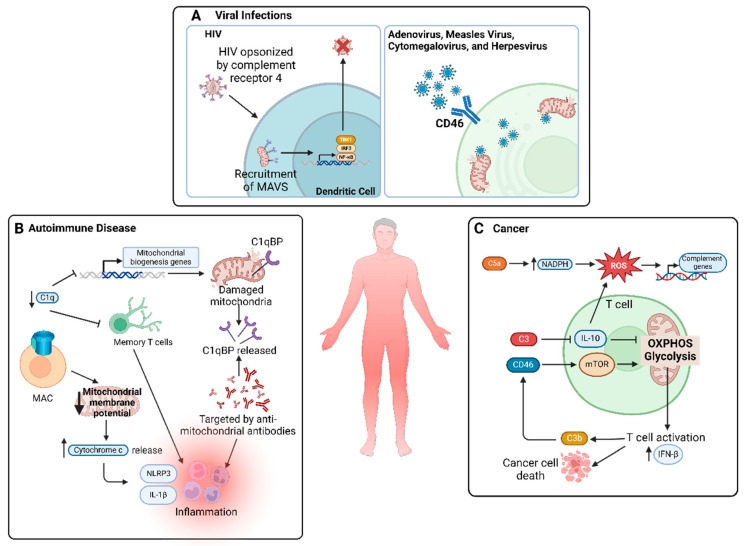
**Interactions between the complement system and mitochondria in systemic diseases.** Complement system and mitochondria interact across various systemic diseases through mitochondrial DAMPs, influencing immune responses and contributing to inflammation, host protection, and defense. (**A**) Human immunodeficiency virus (HIV), when opsonized by complement receptor 4, recruits mitochondrial antiviral-signaling protein (MAVS), triggering the activation of inflammatory pathways, including TANK-binding kinase 1 (TBK1), interferon regulatory factor 3 (IRF3), and nuclear factor kappa B (NF-κB), ultimately aiding elimination of non-opsonized HIV. Several viruses, such as adenovirus, measles virus, cytomegalovirus, herpesvirus, and coxsackievirus utilize CD46 or CD55 as a receptor to enter host cells, resulting in mitochondrial damage. (**B**) C1q deficiency is associated with autoimmune diseases, as C1q can activate mitochondrial biogenesis genes and sustain memory T-cell activity. Functional consequences of C1q deficiency include damaged mitochondria and the release of mitochondrial proteins such as C1qBP, leading to the formation of anti-mitochondrial antibodies and inflammation. Moreover, the formation of membrane attack complex (MAC) in autoimmune diseases has been linked to a lower mitochondrial membrane potential and release of cytochrome c, contributing to NOD-, LRR-, and pyrin domain-containing protein 3 (NLRP3) activation and interleukin (IL)-1β responses. (**C**) In T cells, CD46 can activate the mammalian target of rapamycin (mTOR) and mitochondrial processes such as oxidative phosphorylation (OXPHOS) and glycolysis, both of which are necessary for T-cell activation and subsequent IFN-β response, leading to protective functions of T cells against cancer. Furthermore, C5a has been found to increase ROS by generating NADPH and activating complement genes by oxidative damage.

The interplay between mitochondrial function and complement regulation extends to have broader implications, influencing systemic diseases that span across multiple organ systems. A summary of the cross-talk in systematic diseases is depicted in Figure 4.

### 5.4. Viral Infections

Complement, as a crucial pathway for innate immunity, is undoubtedly important for pathogen clearance in combating viral infections. Its intricate interactions with host cells during such infections involve various components, including complement receptor 4, MAVS, CD46, and CD55.

Human immunodeficiency virus (HIV) caused over half a million deaths and over 35 million infections in 2019 [96]. HIV clearance relies on the interplay between the complement system and MAVS, located on mitochondrial outer membranes. Posch et al. demonstrated that HIV, when opsonized by the complement system and recognized through complement receptor 4, facilitates the recruitment of MAVS [97]. Initiation of the antiviral recognition pathway is further enhanced by opsonized HIV downregulating the MAVS-suppressive RAF-1/PLK1 pathway [97]. Furthermore, they showed that aggregation of MAVS activates the TBK1/IRF3/NF-κB signaling pathway in dendritic cells, a process essential for the elimination of complement-opsonized HIV [97]. Thus, the complement system, in conjunction with MAVS, is essential for the activation of immune responses and effective clearance of HIV, underscoring the importance of mitochondria in complement system-mediated immune activation.

Moreover, complement inhibitor CD46 is known to be an entry receptor for diverse viruses, including adenoviruses, measles virus, cytomegalovirus, and herpes virus [98]. Similarly, complement regulator CD55 acts as a co-receptor for the cardiotropic coxsackievirus B3 [99]. All aforementioned viruses were found to compromise mitochondrial functions, either by disrupting mitochondrial biogenesis or by inducing apoptotic pathways in a mitochondria-mediated fashion [23,100,101,102,103]. As previously discussed, activation of the complement system commonly leads to inhibition of infection; thus, it is likely that CD46, by acting as an inhibitor of the complement system, facilitates viral infections beyond its roles in viral entry. Although no studies have directly linked CD46 with mitochondrial dysfunction during viral infections, the observation that viruses use CD46 as a receptor to invade host cells, leading to subsequent mitochondrial damage, implies that CD46 plays a role in viral mechanisms that impair mitochondrial function. Elucidating whether interactions between viruses and CD46 affect complement regulation and/or mitochondrial function, and understanding the mechanisms involved, is essential in developing treatments to preserve mitochondrial integrity and inhibit viral replication.

In summary, understanding these intricate interactions could help uncover innovative therapeutic interventions targeting both the complement system and mitochondria, offering new avenues to effectively combat viral infections.

### 5.5. Autoimmune Diseases

Autoimmune diseases occur when the immune system erroneously targets the body’s own cells, causing a range of complications such as inflammation and tissue damage. Examples of common autoimmune diseases include systemic lupus erythematosus (SLE), type 1 diabetes, and multiple sclerosis [104]. As part of the immune system, the complement system is a major player in the progression of several autoimmune diseases. However, its involvement in autoimmunity presents a paradox. For example, C1qdeficient individuals are at a higher risk of autoimmune diseases such as SLE and rheumatoid arthritis. Decreases in C1q and formation of anti-C1q-antibodies are observed in patients with SLE [105]. C1q, part of the initiator complex C1 in the classical pathway, along with C1s, C1r, and C4 deficiency (all early complement components), have been associated with SLE [106,107]. Conversely, terminal complement components such as MAC have been implicated in rheumatoid arthritis, glomerulonephritis, and dermatomyositis [106,108,109,110,111,112,113]. Early complement components appear to be decreased or deficient in SLE, while later complement constituents are present. This paradox may be explained by intracellular complement activation such as C3 and C5 activation by CTSL independent of the canonical pathway, allowing for the formation of MAC without early complement components.

The link between mitochondrial DAMPs and autoimmune diseases is underscored by the fact that the release of mitochondrial DAMPs prompts responses from the complement system. For example, the presence of anti-cardiolipin antibodies, prompted by the release of mitochondrial cardiolipin, is a hallmark of SLE and antiphospholipid syndrome/APS [114,115,116]. Notably, the hypothesis that impaired mitochondrial quality control is linked to C1q deficiency gains credence, given the vital role of C1q in the removal of apoptotic cells and immune complexes [52]. Furthermore, C1q is known to activate mitochondrial biogenesis genes and support the maintenance of memory T cells, suggesting a cycle where C1q deficiency leads to mitochondrial dysfunction, which in turn exacerbates immune responses in autoimmunity [50].

This vicious cycle ensues in different ways. Studies have found overproduction of ROS in SLE, rheumatoid arthritis, and multiple sclerosis [117,118,119,120,121,122]. Perl et al. demonstrated increased ROS generation, altered mitochondrial membrane potential, and depleted ATP production in T cells of patients with SLE, indicative of mitochondrial dysfunction [123]. Additionally, Becker et al. reported the release of C1qBP from mitochondrial membranes following mitochondrial damage, which can be targeted by anti-mitochondrial antibodies in SLE [124]. This is important as C1qBP acts as a receptor for C1q and regulates many critical processes such as ATP and ROS generation; thus, the release of C1qBP could potentially lead to dysregulation of ATP and ROS, resulting in mitochondrial dysfunction observed in SLE patients [51,125].

In addition, the presence of MAC in SLE in combination with research findings linking MAC to disruptions in mitochondrial membrane potential suggests another important connection between mitochondria and the complement system in autoimmunity [126]. Specifically, MAC is shown to increase calcium levels and reduce mitochondrial membrane potential, leading to increased cytochrome c release and subsequent increase in NLRP3 inflammasome activation, IL-18 release, and apoptosis [126]. Although the current evidence demonstrates promising links between mitochondrial dysfunction and complement activation, there is a need for more extensive research.

### 5.6. Cancer

Cancer is an ever-growing concern globally, with lung, colorectal, and stomach cancers being the top 3 leading causes of cancer-related deaths worldwide [126]. Cancer cells can divide uncontrollably, evade apoptosis, escape immune detection, and even exploit the immune system to their advantage [127].

T cells are important in cancer immunotherapy research. Notably, the complement system plays a pivotal role in T-cell activation and homeostasis, acting as a mediator bridging innate and adaptive immune responses. Specifically, C3 and C5, which are found in T cells, are activated by a noncanonical protease, CTSL [6,128]. The resulting cleavage fragments, such as C3a and C5a, bind to their respective receptors to regulate immune cell functions [8,129]. Importantly, C3aR and C5aR are located on lysosomal and mitochondrial membranes, contributing to cell homeostasis [9,40,45,46]. This suggests that intracellular complement activity plays a significant role in cancer immune responses, affecting T-cell activation directly or indirectly by potentially altering mitochondrial functions.

In addition to C3, other complement factors have been shown to regulate anti-tumor immunity. For example, activation of complement regulator CD46 was demonstrated to promote the assembly of lysosomal mTOR in T cells, inducing glycolysis and OXPHOS in mitochondria, which are necessary for T-helper cell induction and IFN-β production [10,130,131]. Through its complement regulatory functions, CD46 can also inactivate C3b produced by T cells [6,10,132]. Collectively, these studies underscore a feedback loop involving complement activation, alterations in mitochondrial activity, and T-cell activation. A shift towards OXPHOS and glycolysis to support T-cell induction may result in reduced ATP production, potentially impairing other crucial cellular functions. Further research is needed to unravel the complex interplay between complement systems, mitochondrial dynamics, and T-cell biology.

Beyond the direct effects on anti-tumor immunity, oncogenic cells may induce mitochondrial damage and complement activation through mitochondrial-derived DAMPs. For example, cytochrome c, a mitochondrial DAMP, has been found to be released in breast cancer cells, leading to the induction of immune responses and apoptosis [133,134]. Increased ROS production and mtDNA release have also been observed in cancer cells [135,136,137]. Moreover, C5a has been found to activate ROS-generating NADPH oxidases, resulting in a feedback loop [138,139,140]. Additionally, ROS can induce inflammatory signaling and immune responses, specifically increasing the expression of complement genes [141].

Heat shock proteins (HSPs) also play a role in mitochondrial function and modulating complement response. Mitochondrial HSP70 is expressed highly in tumor cells, and mitochondrial HSP90 exhibits an inhibitory role in C5b-9 or MAC formation [142,143]. This highlights a potential therapeutic direction by targeting HSPs to reduce the immune evasion capabilities of cancer cells. 

## 6. Therapeutic Potential of Targeting Mitochondria and Complement System in Disease Intervention

Mitochondria and the complement system, while established fields of study individually, are understudied areas for therapeutic potential due to the complexity of their interactions and lack of understanding of their connections. The bidirectional nature of this interaction should be noted as a promising avenue for therapeutic intervention. Targeting mitochondria can impact corresponding complement responses, especially through regulation of mitochondrial DAMPs as discussed above, whilst targeting the complement system can both directly and indirectly regulate mitochondrial dysfunction. Currently available drugs, such as eculizumab and pozelimab, target the complement system by inhibiting C5 cleavage [144,145]. Eculizumab has been shown to effectively treat paroxysmal nocturnal hemoglobinuria/PNH and CHAPLE disease, both characterized by a deficiency of negative regulators of complement activation [144,145]. Moreover, C1q presents a potential therapeutic target to address complement–mitochondria interactions in disease progression. For example, the binding of C1q to mitochondrial C1qBP enhanced the expression of mitochondrial biogenesis genes [50]. Drugs targeting C1q, such as C1q inhibitors and anti-C1q antibodies, are currently in the early stages of development [146]. However, further studies are needed to investigate the effect of complement-targeting drugs on mitochondrial function to explore the potential in treating mitochondrial disease, which has limited treatments available.

For interventions directly targeting mitochondria, one of the only existing drugs is antioxidant target ROS to mitigate mitochondrial damage [2]. Studies have shown that antioxidants can restore complement-mediated damage. For example, in cerebral ischemia, antioxidant treatment suppresses C3, reducing neurological damage and infarct volume [147]. Additionally, HSPs represent promising targets, particularly in cancer, where mitochondrial dysfunction and complement dysregulation converge. Particularly, inhibiting HSPs, including mitochondria-localized proteins in the HSP family, leads to strong anti-cancer activity [148]. Increased cytotoxicity of HSP inhibitors may be due to increased complement-mediated cytotoxicity, as HSPs were shown to inhibit MAC formation in cancer cells [143]. Furthermore, modulating CD46 activity in T cells may rescue anti-tumor T-cell responses, which, in combination with other therapeutics, may prove effective in destroying cancer cells [149]. Moreover, targeting specific complement receptors such as C3aR and C5aR holds the potential to modulate inflammatory processes across various diseases. For example, blockade of C5aR improves anti-tumor effects of T cells [150]. However, the efficacy of complement-targeting cancer therapy needs further investigation.

In conclusion, the intersection of mitochondria and the complement system presents a rich landscape for therapeutic innovation. The targeting of multiple components within the complement–mitochondria interaction may yield synergistic effects and overcome the limitations of individual therapies.

## 7. Conclusions

Proven by various studies across different diseases, the complement system and mitochondria are indeed intricately linked. Both systems respond to stress, whereby the release of mitochondrial DAMPs due to stress and physical membrane damage can trigger activation of the complement system. Beyond mitochondrial DAMP signaling, the interaction also includes T-cell activation, direct binding of complement proteins to mitochondrial proteins, and modulation of mitochondrial processes by complement proteins. Furthermore, mitochondrial functions may rely on other essential organelles. These synergistic mechanisms between organelles and the mitochondria–complement system interactions need further research. This bidirectional interplay creates a cycle between the complement system and mitochondria, yet further studies of these interactions may help uncover novel avenues for therapeutic research and identify potential targets for the treatment of various diseases.

## Abbreviations

OXPHOS	Oxidative phosphorylation
DAMP	Damage-associated molecular pattern
ROS	Reactive oxidative species
mtDNA	Mitochondrial DNA
IFN	Interferon
C3	Complement component 3
C5	Complement component 5
C1q	Complement component 1q
iC3b	Inactivated C3b
MAC	Membrane attack complex
GPI	Glycosylphosphatidylinositol
C3aR	C3a receptor
C5aR	C5a receptor
CFH	Complement factor H
CTSL	Cathepsin L
MAVS	Mitochondrial antiviral signaling
IL	Interleukin
C1qBP	C1q-binding protein
I/R	Ischemia/reperfusion
NLRP3	NOD-, LRR-, and pyrin domain-containing protein 3
NF-κB	Nuclear factor kappa B
MBL	Mannose-binding lectin
AD	Alzheimer’s disease
Aβ	Amyloid beta
AMD	Age-related macular degeneration
MI	Myocardial infarction
HIV	Human immunodeficiency virus
SLE	Systemic lupus erythematosus
HSP	Heat shock protein
